# Clinicians’ perceptions of “enhanced recovery after surgery” (ERAS) protocols to improve patient safety in surgery: a national survey from Australia

**DOI:** 10.1186/s13037-024-00397-w

**Published:** 2024-05-23

**Authors:** Josephine Lovegrove, Georgia Tobiano, Wendy Chaboyer, Joan Carlini, Rhea Liang, Keith Addy, Brigid M. Gillespie

**Affiliations:** 1 NHMRC Centre of Research Excellence in Wiser Wound Care, School of Nursing and Midwifery, Griffith University, Gold Coast Campus, 1 Parklands Dr, 4222 Southport, QLD Australia; 2 UQ Centre for Clinical Research, Royal Brisbane and Women?s Hospital, Herston Infectious Diseases Institute, Metro North Health, Level 7, 4029 Herston, Australia; 3 School of Nursing, Midwifery & Social Work, Faculty of Health & Behavioral Sciences, The University of Queensland, 4072 St Lucia, QLD Australia; 4 Gold Coast Hospital & Health Service, Gold Coast University Hospital, 1 Hospital Blvd, 4215 Southport, QLD Australia; 5 Department of Marketing, Griffith University, Gold Coast Campus, 1 Parklands Dr, 4222 Southport, QLD Australia; 6 Gold Coast Health Consumer Advisory Group, Gold Coast Hospital & Health Service, 1 Hospital Blvd, 4215 Southport, QLD Australia; 7 Faculty of Health Sciences, Bond University, 14 University Dr, 4226 Robina, QLD Australia; 8 Robina Hospital, Gold Coast Hospital & Health Service, 2 Bayberry Ln, 4226 Robina, QLD Australia

**Keywords:** Clinical protocols, Critical pathways, General surgery, Guideline adherence, Intraoperative complications, Operating rooms, Postoperative complications, Surgical procedures, operative

## Abstract

**Background:**

Surgical patients are at risk of postoperative complications, which may lead to increased morbidity, mortality, hospital length-of-stay and healthcare costs. Enhanced Recovery After Surgery (ERAS®) protocols are evidence-based and have demonstrated effectiveness in decreasing complications and associated consequences. However, their adoption in Australia has been limited and the reason for this is unclear. This study aimed to describe clinicians’ perceptions of ERAS protocols in Australia.

**Methods:**

A national online survey of anaesthetists, surgeons and nurses was undertaken. Invitations to participate were distributed via emails from professional colleges. The 30-item survey captured respondent characteristics, ERAS perceptions, beliefs, education and learning preferences and future planning considerations. The final question was open-ended for elaboration of perceptions of ERAS. Descriptive and inferential statistics were used to describe and compare group differences across disciplines relative to perceptions of ERAS.

**Results:**

The sample included 178 responses (116 nurses, 65.2%; 36 surgeons, 20.2%; 26 anaesthetists, 14.6%) across six states and two territories. More than half (*n* = 104; 58.8%) had used ERAS protocols in patient care, and most perceived they were ‘very knowledgeable’ (*n* = 24; 13.6%) or ‘knowledgeable’ (*n* = 71; 40.3%) of ERAS. However, fewer nurses had cared for a patient using ERAS (*p* <.01) and nurses reported lower levels of knowledge (*p* <.001) than their medical counterparts. Most respondents agreed ERAS protocols improved patient care and financial efficiency and were a reasonable time investment (overall *Md* 3–5), but nurses generally recorded lower levels of agreement (*p.*013 to < 0.001). Lack of information was the greatest barrier to ERAS knowledge (*n* = 97; 62.6%), while seminars/lectures from international and national leaders were the preferred learning method (*n* = 59; 41.3%). Most supported broad implementation of ERAS (*n* = 130; 87.8%).

**Conclusion:**

There is a need to promote ERAS and provide education, which may be nuanced based on the results, to improve implementation in Australia. Nurses particularly need to be engaged in ERAS protocols given their significant presence throughout the surgical journey. There is also a need to co-design implementation strategies with stakeholders that target identified facilitators and barriers, including lack of support from senior administration, managers and clinicians and resource constraints.

**Supplementary Information:**

The online version contains supplementary material available at 10.1186/s13037-024-00397-w.

## Background

Globally, the demand for surgery is high; around 313 million procedures were performed in 2012 [1] and this figure is likely now far higher [2]. Due to the invasive nature of surgery, patients are at risk of developing postoperative complications. A global study of patient outcomes after elective surgery across 474 hospitals in 27 countries found that 16.8% of 44,814 patients developed complications postoperatively, including surgical site infection (5%), bleeding (3%), arrhythmia (2.7%) and pneumonia (1.6%) [3]. The impacts of such postoperative complications can be far-reaching. For instance, they may result in psychological consequences [4], decreased quality of life [5], increased hospital length of stay and costs [6, 7] and mortality [3]. Globally, four million individuals die within 30-days of surgery annually, which has been estimated to represent 7.7% of global deaths [2]. Subsequently, best-practice surgical care pathways, guidelines, and protocols have been developed to reduce patients’ risk of developing postoperative complications.

Enhanced Recovery After Surgery (ERAS®) protocols were developed based on evidence to reduce perioperative stress and complications and promote early recovery [8, 9]. A growing body of research has demonstrated the positive impacts of using ERAS protocols compared to traditional care, and there have been several systematic reviews, meta-analyses and an umbrella review supporting the effectiveness of ERAS protocols on reducing hospital length of stay, costs, readmissions and/or complication rates [e.g. 10-13]. First created for colon resection [14], ERAS protocols have now been adapted and updated for multiple surgical specialties (e.g., elective colorectal surgery [15], cardiac surgery [16]) and specific procedures (e.g., pancreaticoduodenectomy [17]). They span the entire surgical journey; and are intended to be multimodal, requiring a multidisciplinary approach with no single element alone improving patient outcomes [9]. Specific protocols vary depending on the type of procedure, preoperative components include education, lifestyle changes, nutritional support, dietary interventions, and medication use [15, 16–17]. The intraoperative phase includes temperature control, medication use, and minimisation of invasiveness and drains. Postoperatively, patients receive multimodal opioid-sparing pain control, early drain removal, mobilisation, oral fluid intake, and follow-up. Prophylactic measures (e.g., anti-emesis, -microbial or -coagulation) are also included across surgical protocol phases.

However, despite the benefits of ERAS protocol use [13], their free availability [8] and their uptake internationally, wider adoption in the Australian context has been slow or incomplete [18]. There is a myriad of reasons for this, including the complexity of ERAS implementation and its resource-intensive nature. Internationally, barriers to ERAS protocol implementation include resistance to change from frontline clinicians, limited implementation resources, and external factors (e.g., patient complexity, rural location) [19]. Another key barrier in Australia may be the fragmentation of care delivery between different phases of the surgical journey such as preadmission versus perioperative phases versus surgical ward care [18]. Selection and prioritisation of only some components as opposed to implementation of a protocol in its entirety is another challenge in ERAS implementation [20, 21], which may result for reasons such as a lack of surgery- or specialty-specific ERAS protocols in some areas.

Compounding these barriers has been the lack of a nationally coordinated approach to ERAS implementation and compliance monitoring in Australia, as opposed to the governmental support observed in Canada, the United Kingdon, New Zealand and the United States [20]. National approaches to drive sustained ERAS implementation in such countries are only recently emerging, including the establishment of a national ERAS Centre of Excellence [22] and state-based government guidance and support [23–25]. This means that ERAS implementation in Australia remains in the early stages, with the impetus for implementation previously dependent upon individual motivated clinicians and facilities. At the clinician level, awareness and agreement with clinical practice guidelines precedes their adoption and adherence [26, 27]. However, the extent of clinicians’ perceptions of ERAS protocols in Australia is unknown. A recent study examined colorectal surgeons’ attitudes towards ERAS interventions in Australia and New Zealand [28], but this was limited to rating the perceived effectiveness of individual ERAS components and other clinicians were not included. A broader investigation may reveal important insight into the lagging adoption of ERAS protocols. There is a need to explore this knowledge gap to inform the development of strategies to promote and enable more consistent implementation of ERAS protocols in the Australian context.

### Aim

This study aimed to describe and compare clinicians’ (anaesthetists, surgeons and nurses) perceptions of ERAS protocols in Australia. Subsumed in this overarching aim were the following objectives:

1. Describe and compare anaesthetists’, surgeons’ and nurses’ demographic characteristics and previous ERAS care experience.
2. Describe and compare anaesthetists’, surgeons’ and nurses’ perceived knowledge and beliefs about ERAS protocols.
3. Describe and compare anaesthetists’, surgeons’ and nurses’ learning and education preferences and interests about ERAS protocols.
4. Describe and compare anaesthetists’, surgeons’ and nurses’ support for ERAS implementation.
5. Describe clinicians’ overall perceptions of ERAS protocols including barriers and facilitators for their use.

## Methods

### Design

A descriptive national online survey of surgeons, anaesthetists, and perioperative and surgical nurses was undertaken. Its reporting was guided by the Checklist for Reporting of Survey Studies (CROSS, Supplementary file S1) [29]. The involvement of consumers as co-investigators and partners in conducting the survey was guided by the principles of consumer partnerships in research [30] and reported using the Guidance for Reporting Involvement of Patients and the Public short-form checklist (GRIPP 2; Supplementary file S2) [31].

### Sample

The target population was healthcare professionals from surgery, anaesthetics, perioperative and surgical nursing who were currently practicing in Australia. The sample was drawn from fellows and members of four professional organisations; the Royal Australasian College of Surgeons (RACS, ≈ 6840 active Fellows); the Australian Society of Anaesthetists (ASA, ≈ 3400 active members); the Australian College of Perioperative Nurses (ACORN, ≈ 5500 active members); and the Australian College of Nursing (ACN, ≈ 7200 active members). Despite the Australian and New Zealand College of Anaesthetists’ (ANZCA, ≈ 8000 active members) initial agreement to disseminate the survey via email, this did not occur. Table 1 shows the eligibility criteria.

**Table 1:** Eligibility criteria

• Anaesthetists and surgeons currently practicing in Australia in any specialty.
• Nurses practicing in Australia in any perioperative role (e.g., instrument/circulation, perianaesthesia, post-anaesthetic recovery) or surgical setting (e.g., surgical ward, preadmission clinic, post-surgical outpatient).

### Survey instrument

The study survey instrument was adapted from Beal et al’s [32]. original survey. The survey intended to elicit surgery and anaesthesia providers (including surgeons, anaesthetists, postgraduate trainees, advanced practice nurses) knowledge and preferences for learning about ERAS protocols. The original survey was developed in the United States and was adapted to the Australian context for this study. Minor adaptations included word changes to professional role (e.g., changed from “Attending” to “Consultant Surgeon/Anaesthetist/Registrar Surgeon/Anaesthetist” and “CRNA” to “Perioperative Nurse”). Item additions included the type of operating rooms in respondents’ practice facility (e.g., “Outpatient/Inpatient/Adult/Paediatric”), the healthcare sector in which respondents practiced (e.g., public/Private), location of facility (e.g., “Country/City/State/Territory”), and the types of surgical teams respondents worked in (e.g., general surgery, urology, orthopaedics). We also asked respondents their age and the number of years of clinical experience as continuous variables rather than treating them as categorical variables as was done in the original survey. The adapted survey comprised 28 items across four sections measuring (1) ***demographics***, (2) ***perceptions of ERAS***, (3) ***knowledge of ERAS (education and learning preferences)*** and (4) ***future planning*** for ERAS (see Supplementary file S3).

The ***demographics*** section had 14 items including age; sex; location; healthcare sector, type of facility, department, specialty and unit of work; current role and years in role. Department, specialty and role items were nuanced towards either nurses or anaesthetists and surgeons (see Supplementary file S3). A screening question was also included at the beginning of the nurse survey; “Do you currently work in a setting that provides post-operative surgical care?” (yes/no; if no survey ended). Additionally, if any participant’s response indicated their current facility was not located in Australia, the survey ended. Finally, the demographics section measured whether respondents had participated in the care of a patient using an ERAS protocol previously. ***Perceptions of ERAS*** included seven items with responses measured on a 5-point Likert scale to indicate participants’ degree of perceived knowledge (1 = Very unknowledgeable, 5 = Very knowledgeable) and their beliefs of the benefits of ERAS (1 = Strongly Disagree; 5 = Strongly Agree). ***Knowledge of ERAS*** focused on ***education and learning*** and had five items with multiple choice questions relating to reasons for ERAS design, interest in and preferred method of ERAS learning, need for formal ERAS education for each profession, and barriers to gaining ERAS knowledge. Finally, the ***future planning*** section had two items, one measuring whether ERAS protocols should be implemented broadly and if so, for which populations, and one free-text item for additional comments or thoughts.

### Data collection

Permission to access prospective survey respondents was granted by professional organisations that distributed the survey to fellows and members on behalf of the researchers via their regular email communications. Emails were sent to all active fellows and members (≈ 22,940 total), although it was unclear how many members and fellows were eligible to respond. Emails contained a brief invitation and link, which guided respondents to the participant information sheet, followed by the survey, all hosted within the Research Data Capture (REDCap) data management system [33]. Respondents were able to exit the survey at any time by closing their browser. The online platform used to host the survey recorded all data entered in the survey, including incomplete responses. Therefore, if a participant provided consent but did not fully complete the survey, their data was retained for analysis.

Recruitment was active from July to November 2023. After the initial invitation was sent to organisation fellows and members, with two follow-up invitations sent. Given the large number of fellows and members, and distribution of the invitation via professional organisations, there was no individual or identifiable tracking of who received, opened, or completed the survey. Several members of the research team also regularly posted invitation links to the survey on X, Inc™.

### Data analysis

Data were exported from REDCap into Microsoft Excel™ for cleaning, and response rates calculated. Data were then imported into IBM SPSS™ (Version 29) for further analysis. Descriptive statistics were used to summarise the data, with frequencies (counts and proportions) used to report categorical data. Summary statistics using means and standard deviations or medians and interquartile ranges (IQR) were used for continuous data, depending on their distribution. Ordinal 5-point Likert scale responses were described using counts and proportions, and an overall median, interquartile range and range. Differences between the three professional disciplines were examined using Pearson Chi-square, Fisher’s Exact, one-way between-groups analysis of variance, or Kruskal-Wallis tests, where appropriate and based on assumptions. Pearson Chi-square (with Yates Correction for 2 × 2 tables) tests were used to compare associations between perceptions of ERAS and nursing departments (perioperative and surgical ward/unit). Statistical significance was set at *p* <.05.

## Results

### Survey responses

**Fig. 1:**
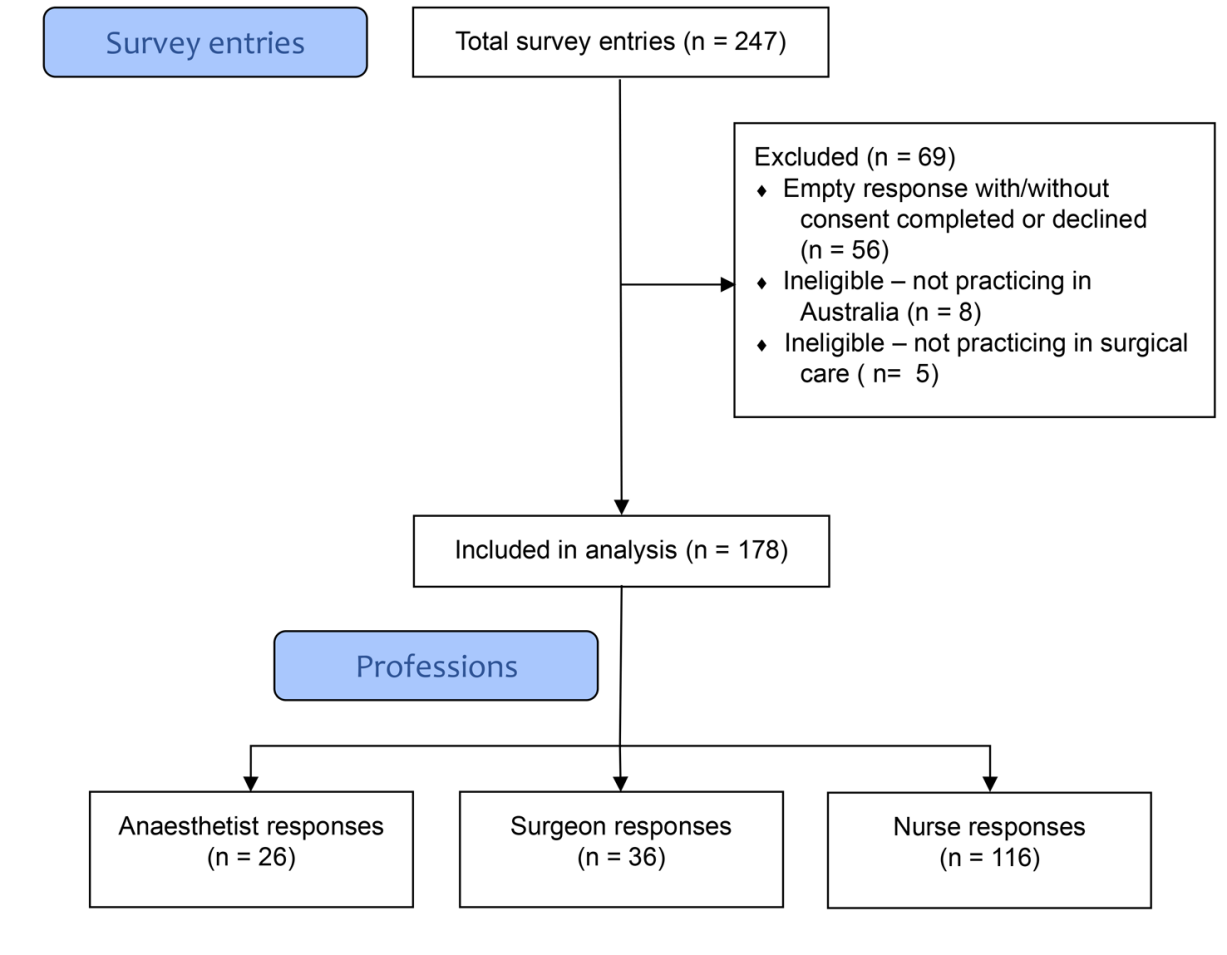
Survey entries, losses, and responses [after 34]

A total sample of 178 was included, comprising 116 (65.2%) nurses, 36 (20.2%) surgeons and 26 (14.6%) anaesthetists (see Fig. 1). Of these, 23 (12.9%) had large amounts of missing data, with these respondents completing the survey to the end or beginning of the ‘Demographics’ and ‘Perceptions of ERAS’ sections, respectively. Cases of missing data are reported in the results tables.

### Sample characteristics (objective 1)

Table 2 describes characteristics of the sample. Respondents were located across six states and two territories, with nurse and surgeon respondents in all. There were significant differences across professions in terms of sex, adult and paediatric operating theatres in respondents’ facilities and specialties worked within (Table 2). Over three-quarters of nurses were perioperative as opposed to surgical. Almost 60% of respondents reported previously participating in the care of a patient who was on an ERAS protocol. There was a significant association between reports of previously participating in the care of a patient who was on an ERAS protocol and profession (χ^2^ [2, *n* = 177] = 40.96, *p* <.01, *Cramer’s V* = 0.48; Table 2). These results indicated that fewer than 50% of nurses had participated previously, while all anesthetists and over 80% of surgeons had previously participated in ERAS protocols.

**Table 2:** Sample demographics

	Overall *n* = 178 (100%)	Anaesthetists *n* = 26 (14.6%)	Surgeons *n* = 36 (20.2%)	Nurses *n* = 116 (65.2%)	*p* value
Age* mean (SD)	47.4 (11.3)	42.8 (8.4)	49.1 (12.5)	48.0 (11.3)	0.074
Sex* ***n*** **(%)**					
Female	120 (68.2%)	10 (38.5%)	9 (25.0%)	101 (88.6%)	
Male	55 (31.3%)	16 (61.5%)	27 (75.0%)	12 (10.5%)	< 0.001
Prefer not to answer	1 (0.6%)	0 (0.0%)	0 (0.0%)	1 (0.9%)	
**Sector** ***n*** **(%)**					
Public	130 (73.0%)	22 (84.6%)	27 (75.0%)	81 (69.8%)	0.303
Private	48 (27.0%)	4 (15.4%)	9 (25.0%)	35 (30.2%)	
Location* ***n*** **(%)**					
Victoria	48 (27.7%)	4 (15.4%)	13 (37.1%)	31 (27.7%)	
New South Wales	47 (27.2%)	8 (30.8%)	5 (14.3%)	34 (30.4%)	
Queensland	38 (22.0%)	7 (26.9%)	6 (17.1%)	25 (22.3%)	
Western Australia	16 (9.2%)	4 (15.4%)	4 (11.4%)	8 (7.1%)	0.438
South Australia	15 (8.7%)	3 (11.5%)	3 (8.6%)	9 (8.0%)	
Australian Capital Territory	4 (2.3%)	0 (0.0%)	2 (5.7%)	2 (1.8%)	
Tasmania	3 (1.7%)	0 (0.0%)	1 (2.9%)	2 (1.8%)	
Northern Territory	2 (1.2%)	0 (0.0%)	1 (2.9%)	1 (0.9%)	
**Type of ORs at facility** ***n*** **(%)**					
Inpatient surgery	161 (90.4%)	23 (88.5%)	35 (97.2%)	103 (88.8%)	0.324
Adult surgery	158 (88.8%)	23 (88.5%)	27 (75.0%)	108 (93.1%)	0.011
Outpatient surgery	107 (60.1%)	17 (65.4%)	23 (63.9%)	67 (57.8%)	0.690
Paediatric surgery	94 (52.8%)	14 (53.8%)	9 (25.0%)	71 (61.2%)	< 0.001
**Department** ***n*** **(%)**	NA	Anaesthesia 25 (96.2%)	Surgery 36 (100%)	Perioperative 99 (85.3%)	NA
		Surgery 1 (3.8%)		Surgical ward/unit 17 (14.7%)	
Surgeon/Anaesthetist specialty* ***n*** **%**	NA	Generalist 9 (69.2%)	Colorectal 5 (33.3%)	NA	NA
		Cardiac 3 (23.1%)	Urology 3 (20.0%)		
		Upper GI 1 (7.7%)	General Surgery 2 (13.3%)		
			Orthopaedic 2 (13.3%)		
			OG 1 (6.7%)		
			Thoracic 1 (6.7%)		
			Plastics 1 (6.7%)		
Surgical team/service* ***n*** **(%)**					
General Surgery	131 (73.6%)	24 (92.3%)	16 (44.4%)	91 (78.4%)	< 0.001
Orthopaedics	102 (57.3%)	20 (76.9%)	5 (13.9%)	77 (66.4%)	< 0.001
Plastics	89 (50.0%)	18 (69.2%)	5 (13.9%)	66 (56.9%)	< 0.001
Urology	89 (50.0%)	19 (73.1%)	6 (16.7%)	64 (55.2%)	< 0.001
Gynaecology	87 (48.9%)	14 (53.8%)	2 (5.6%)	71 (61.2%)	< 0.001
Ears Nose Throat	78 (43.8%)	16 (61.5%)	2 (5.6%)	60 (51.7%)	< 0.001
Ophthalmology	57 (32.0%)	4 (15.4%)	0 (0.0%)	53 (45.7%)	< 0.001
Dental/Oral Surgery	55 (30.9%)	6 (23.1%)	0 (0.0%)	49 (42.2%)	< 0.001
Trauma	51 (28.7%)	11 (42.3%)	4 (11.1%)	36 (31.0%)	0.17
Vascular	50 (28.1%)	13 (50.0%)	2 (5.6%)	35 (30.2%)	< 0.001
Thoracic	31 (17.4%)	6 (23.1%)	1 (2.8%)	24 (20.7%)	0.019
Neurosurgery	27 (15.2%)	10 (38.5%)	0 (0.0%)	17 (14.7%)	< 0.001
Cardiac	16 (9.0%)	3 (11.5%)	0 (0.0%)	13 (11.2%)	0.071
Transplant	10 (5.6%)	3 (11.5%)	0 (0.0%)	7 (6.0%)	0.106
Burns	8 (4.5%)	2 (7.7%)	0 (0.0%)	6 (5.2%)	0.250
Other	25 (14.0%)	5 (19.2%)	2 (5.6%)	18 (15.5%)	0.204
Role* ***n*** **(%)**	NA	Consultant Anaesthetist	Consultant Surgeon 16 (44.4%)	***Perioperative*** C/I RN; surgical assistant 35 (35.7%)	NA
		20 (76.9%)	Registrar Surgeon	Anaesthetics; PACU RN 24 (24.5%)	
		Registrar Anaesthetist	6 (16.7%)	NUM 15 (15.3%)	
		4 (15.4%)	Surgical Fellow	NE 10 (10.2%)	
		Anaesthetic Fellow	2 (5.6%)	CNC 3 (3.1%)	
		1 (3.8%)	Unspecified	CN 2 (2.0%)	
		Unspecified 1 (3.8%)	12 (33.3%)	DON; ADON 2 (2.0%)	
				RN 2 (2.0%)	
				ERAS® coordinator 1 (1.0%)	
				Multi-role (C/I, anaesthetics, PACU, day surgery and/or surgical ward) 4 (4.1%)	
				***Surgical ward/unit***	
				RN 15 (88.2%)	
				NE 1 (5.9%)	
				NUM 1 (5.9%)	
Years in current role* median (range)	10 (0.5–54)	7.5 (1–35)	13 (1–40)	9 (0.5–54)	0.183
I have participated in the care of a patient who was on an ERAS protocol* ***n*** **(%)**	104 (58.8%)	26 (100%)	30 (83.3%)	48 (41.7%)	< 0.001

*Note:* C/I = Circulating/Instrument; CNC = Clinical Nurse Consultant; ERAS = Enhanced Recovery After Surgery; GI = Gastrointestinal; NA = not applicable; NE = Nurse Educator; NUM = Nurse Unit Manager; OG = Obstetrics & Gynaecology; OR = Operating Rooms; PACU = Post-Anaesthetic Care Unit; RN = Registered Nurse; SD = standard deviation. *Missing data 7.3%, 1.1%, 2.8%, 19.1%, 0.6%, 0.6%, 0.6%, 1.1%, 0.6%, respectively. Other = obstetrics, endoscopy, pain management, ECT, gastroenterology/hepatobiliary, bariatric, colorectal, interventional radiology, neurovascular/cardiac repair prior to tertiary referral, podiatric, staff education/waitlist, undefined

### Perceptions of ERAS (objective 2)

#### Knowledge level

**Fig. 2:**
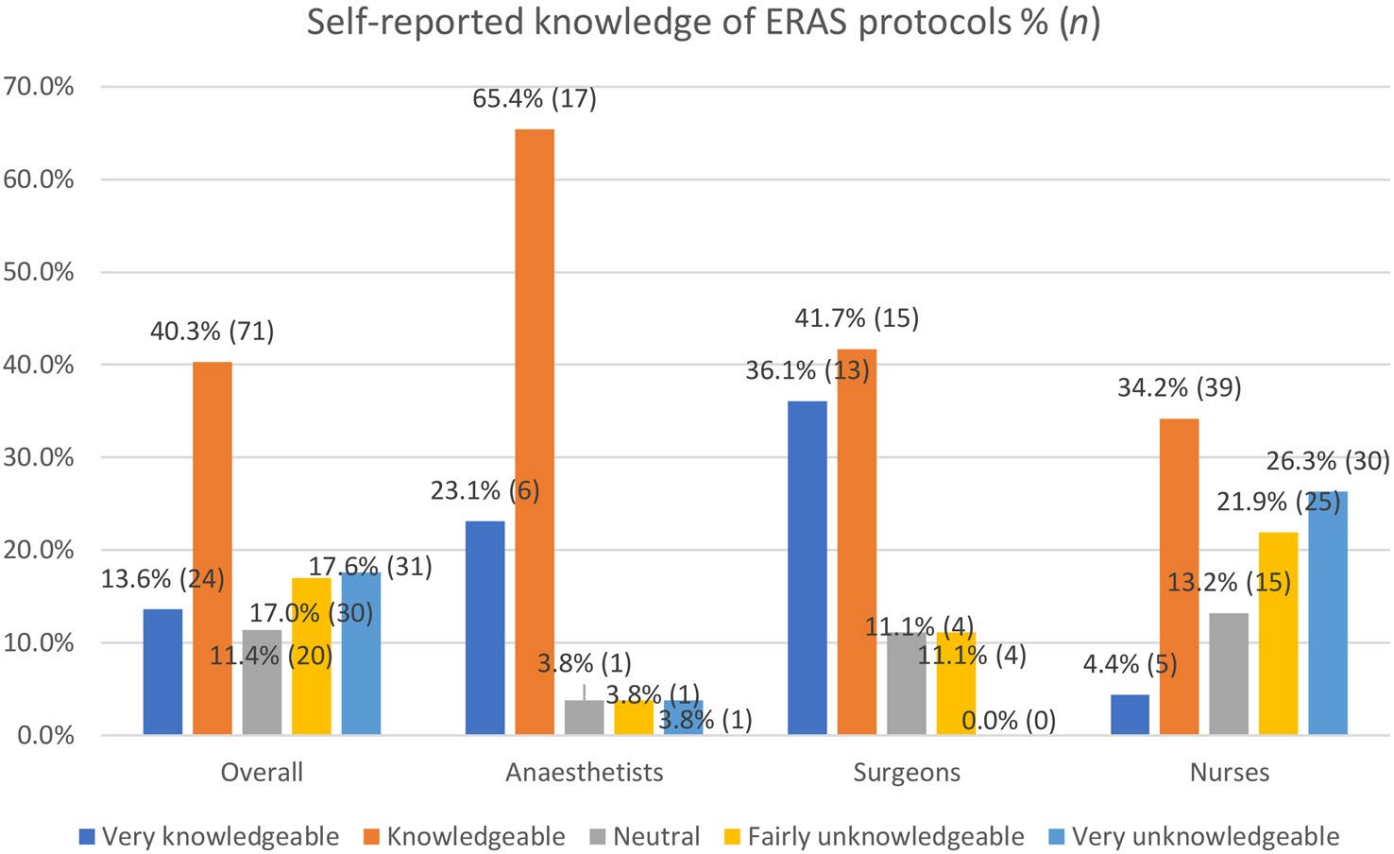
Respondent knowledge levels

Over half of respondents rated their knowledge level as ‘knowledgeable’ or ‘very knowledgeable’ (Fig. 2; *Md* = 4, IQR = 2, range 1–5). Differences in knowledge levels across groups were statistically significant ([Gp1, *n* = 26: Anaesthetists, Gp2, *n* = 36: Surgeons, Gp3, *n* = 114: Nurses], χ^2^ [2, *n* = 176] = 41.25, *p* <.001). Post hoc pairwise correction using Bonferroni correction indicated nurses had a significantly lower median score (*Md* = 3, IQR = 3, range 1–5) compared to anaesthetists (*Md* = 4, IQR = 0, range 1–5, *p* <.001) and surgeons (*Md* = 4, IQR = 1, range = 2–5, *p* <.001), while differences between the latter two groups were not significant (*p* = 1.0). Perceived knowledge level was not associated with nurses’ department (perioperative and surgical ward/unit).

### Beliefs about ERAS

The level of agreement about the benefits of using ERAS among respondents was neutral to strongly agree. Most respondents perceived that ERAS protocols improve patient care and institution financial efficiency and are a reasonable time investment (Supplementary file S4), with overall medians ranging from 3/5 to 5/5 across statements. There were significant differences between professions for most statements (*n* = 5), with nurses generally recording lower levels of agreement (Table 3; Supplementary file S4). Pairwise post hoc comparisons suggested that differences between surgeons and anaesthetists were not significant for any statements. However, there were significant differences between nurses and anaesthetists (*n* = 1), surgeons (*n* = 1) or both (*n* = 3) (Table 3). Statement scores were not associated with nurses’ department (perioperative and surgical ward/unit).

**Table 3:** Perceptions of ERAS: Median (IQR, range)

	Overall *n* = 178 (100%)	Anaesthetists *n* = 26 (14.6%)	Surgeons *n* = 36 (20.2%)	Nurses *n* = 116 (65.2%)	p value
I believe ERAS® protocols improve patient care*n (%)	4 (1, 2–5)	5 (1, 4–5)	5 (1, 2–5)	4 (1, 3–5)	<.001^a^
I believe the hospital administration thinks ERAS® protocols improve patient care*n (%)	3 (1, 2–5)	4 (1, 3–5)	4 (1, 2–5)	3 (1, 2–5)	0.364
I believe my colleagues think ERAS® protocols improve patient* care*n* (%)	4 (1, 2–5)	4 (0, 2–5)	4 (1, 2–5)	3 (1, 2–5)	.013^b^
I believe that my patients have/will have improved care when they are involved in ERAS® protocols*n (%)	4 (1, 2–5)	4 (1, 2–5)	4 (2, 2–5)	4 (1, 2–5)	.004^a^
I believe ERAS® protocols are a reasonable investment of my time* *n* (%)	4 (2, 2–5)	4 (1, 3–5)	4 (1, 2–5)	4 (1, 2–5)	<.001^a^
I believe that ERAS® protocols improve/will improve the financial efficiency of our institution* *n* (%)	4 (2, 2–5)	4 (1, 3–5)	4.5 (1, 2–5)	4 (1, 2–5)	<.001^c^

*Note:*^a^ pairwise: significant difference between nurses versus surgeons/anaesthetists; ^b^pairwise: significant difference between nurses versus anaesthetists; ^c^pairwise: significant difference between nurses versus surgeons; ERAS = Enhanced Recovery After Surgery; IQR = interquartile range. Missing data* 12.9%, 14%, 14%, 12.9%, 12.9%, 14%

### Knowledge (education and learning preferences) of ERAS protocols (objective 3)

Table 4 summarises participants’ responses to the ‘Knowledge of ERAS’ survey component. Respondents were most interested in learning about minimising perioperative complications and least interested in learning about improving perioperative efficiency, with significant associations between profession and interest in learning about fluid management and minimising perioperative complications (Table 4). Post hoc pairwise comparisons using Bonferroni correction indicated significant differences between nurses and anaesthetists for both (*p* =.003 and *p* =.001, respectively). Most respondents indicated their preferred method to learn about ERAS® was via seminars or lectures from national or international leaders overall and across professions (Table 4). However, more surgeons indicated they would prefer to learn via direct participation in protocols and reviewing journal articles or textbooks. Post hoc group comparisons indicated group differences were significant (*n* = 143, *p* =.031; Table 4). Nearly all respondents supported formal education on ERAS® for upcoming nurses, followed by training for surgeons and anaesthetists. However, significantly fewer anaesthetists supported such training for upcoming practitioners in their profession. More nurses than anaesthetists and surgeons perceived that lack of information was a barrier to gaining ERAS knowledge (χ^2^ [2, *n* = 155] = 10.33, *p* =.006, *Cramer’s V* =.258).

**Table 4:** Knowledge of and future planning for ERAS

Knowledge of ERAS protocols			Overall *n* = 178 (100%)	Anaesthetists *n* = 26 (14.6%)	Surgeons *n* = 36 (20.2%)	Nurses *n* = 116 (65.2%)	*p* value
ERAS protocols are primarily designed to* ***n*** **(%)**	a. Reduce the patient’s response to surgical stress to improve length of stay and reduce postoperative complications and mortality		26 (16.9%)	4 (16.0%)	8 (25.0%)	14 (14.4%)	0.619
	b. Enhance the hospital’s efficiency and result in better financial outcomes for the hospital		6 (3.9%)	1 (4.0%)	1 (3.1%)	4 (4.1%)	
	c. Address patient expectations preoperatively to lead to improved patient satisfaction		6 (3.9%)	0 (0.0%)	0 (0.0%)	6 (6.2%)	
	d. All the above		116 (75.3%)	20 (80.0%)	23 (71.9%)	73 (75.3%)	
**I am most interested in learning more about…** ***n*** **(%)**	Fluid Management*	1: Most interested	17 (13.4%)	2 (10.0%)	4 (15.4%)	11 (13.6%)	0.001^a^
		2	28 (22%)	12 (60.0%)	7 (26.9%)	9 (11.1%)	
		3	37 (29.1%)	4 (20.0%)	10 (38.5%)	23 (28.4%)	
		4: Least interested	45 (35.4%)	2 (10.0%)	5 (19.2%)	38 (46.9%)	
	Multimodal Pain Management*	1: Most interested	36 (27.9%)	11 (55.0%)	6 (25.0%)	19 (22.4%)	0.208
		2	37 (28.7%)	2 (10.0%)	6 (25.0%)	29 (34.1%)	
		3	40 (31.0%)	4 (20.0%)	7 (29.2%)		
		4: Least interested	16 (12.4%)	3 (15.0%)	5 (20.8%)	29 (34.1%)	
						8 (9.4%)	
	Minimising Perioperative Complications*	1: Most interested	48 (37.5%)	3 (13.0%)	9 (36.0%)	36 (45.0%)	0.001^a^
		2	38 (29.7%)	5 (21.7%)	6 (24.0%)	27 (33.8%)	
		3	29 (22.7%)	12 (52.2%)	5 (20.0%)		
		4: Least interested	13 (10.2%)	3 (13.0%)	5 (20.0%)	12 (15.0%)	
						5 (6.3%)	
	Improving Perioperative Efficiency*	1: Most interested	23 (16.3%)	3 (13.0%)	4 (13.8%)	16 (18.0%)	0.375
		2	30 (21.3%)	4 (17.4%)	8 (27.6%)	18 (20.2%)	
		3	26 (18.4%)	2 (8.7%)	5 (17.2%)		
		4: Least interested	62 (44.0%)	14 (60.9%)	12 (41.4%)	19 (21.3%)	
						36 (40.4%)	
My preferred method to learn about ERAS is* ***n*** **(%)**	a. Direct participation in institutional protocols		32 (22.4%)	3 (13.6%)	8 (26.7%)	21 (23.1%)	0.031
	b. Reviewing journal articles or textbooks		18 (12.6%)	5 (22.7%)	8 (26.7%)	5 (5.5%)	
	c. Seminars or lectures on the topic from leaders at national or international levels		59 (41.3%)	9 (40.9%)	10 (33.3%)	40 (44.0%)	
	d. Seminars or lectures on the topic from leaders within my hospital or unit		34 (23.8%)	5 (22.7%)	4 (13.3%)	25 (27.5%)	
**I think formal education about ERAS should be part of training for upcoming…** ***n*** **(%)**	Anaesthetists*		126 (87.5%)	14 (70.0%)	24 (82.8%)	88 (92.6%)	0.012
	Surgeons*		133 (93.0%)	19 (95.0%)	26 (89.7%)	88 (93.6%)	0.705
	Nurses*		143 (99.3%)	20 (100%)	29 (100%)	94 (98.9%)	*1.0*
I think barriers to gaining knowledge about ERAS include* ***n*** **(%)**	Lack of information provided		97 (62.6%)	14 (56.0%)	13 (40.6%)	70 (71.4%)	0.006
	Lack of interest from providers		77 (49.7%)	14 (56.0%)	17 (53.1%)	46 (46.9%)	0.677
	Lack of time		76 (49.0%)	12 (48.0%)	11 (34.4%)	53 (54.1%)	0.156
	Lack of resources		72 (46.5%)	12 (48.0%)	15 (46.9%)	45 (45.9%)	1.00
	Lack of research		19 (12.3%)	1 (4.0%)	2 (6.3%)	16 (16.3%)	0.157
	Lack of interest from patients		18 (11.6%)	2 (8.0%)	3 (9.4%)	13 (13.3%)	0.817

*Note*^a^pairwise: significant difference between nurses versus anaesthetists; ERAS = Enhanced Recovery After Surgery. *Missing data 13.5%, 28.7%, 27.5%, 28.1%, 20.8%, 19.1%, 19.7%, 19.1%, 12.9%, respectively

### Future planning (objective 4)

Most respondents supported the broad implementation of ERAS protocols (*n* = 130, 87.8%). Support was similar across professions (anaesthetists *n* = 22, 100%; surgeons *n =* 28, 90.3%; nurses *n* = 80, 84.2%; *n* = 148, *p* =.107). Of those that supported implementation of ERAS protocols, 86 (66.2%%) provided further comment on the populations where ERAS should be implemented. Of those 86, supported groups for implementation were all or almost all patients (*n* = 47, 54.7%), all adults (*n* = 5, 5.8%), major and/or complex operations (*n* = 11, 12.8%), specific specialties (*n* = 7, 8.1%), specific vulnerable populations (*n* = 7, 8.1%), elective operations and patients (*n* = 4, 4.7%), day surgery (*n* = 1, 1.2%), post-operatively (*n* = 1, 1.2%), and for those admitted to the ward post-operatively (*n* = 1, 1.2%), with suitable carers and home environments (*n* = 1, 1.2%) and workers and carers (*n* = 1, 1.2%). Two further respondents (1.54%) supported implementation of ERAS protocols but indicated that they felt its use was not appropriate for specific patient groups or that use should be selective based on patient circumstances.

Of those that did not support broad ERAS implementation, 44.4% (*n* = 8/18) provided further comment. Four specified that they either did not know enough about ERAS to answer (50%), while the remaining four specified that only certain ERAS principles were relevant, that it depends on the surgery and patient, that nurses lose critical thinking skills and knowledge due to everything becoming ‘ticking a box’, or that protocols are difficult to implement during short pre-operative periods, in limited length of stay procedures and within pre-existing local procedures.

### Further comments (objective 5)

In addition to the comments presented in the previous section, there were a further 49 survey comments. Comments ranged from detailing local practices that may already incorporate components of ERAS, ‘selective’ implementation and benefits of ERAS to the identification of barriers and facilitators. Several respondents indicated they or their colleagues had limited or no awareness of ERAS protocols (*n* = 10, 5.62%).

### Barriers and facilitators to ERAS implementation

There were several barriers and facilitators to ERAS implementation identified in the comments, (Table 5). Major barriers related to lack of knowledge and understanding of benefits, lack of interest and support from senior administration, managers, and clinicians for implementation and standardisation, resource requirements and a lack of ability to adapt content based on patient needs.

**Table 5:** Barriers and facilitators to ERAS implementation

Barriers	Facilitators
***Lack of knowledge and seniority support*** • Poor understanding / experience / interest in ERAS among senior clinicians and leaders • Resistance to change/moving away from historical models • Lack of understanding of long-term benefits versus up-front investment • High nursing turnover on wards and required education for staff rotations • Limited and non-compulsory implementation at a department/hospital level and institutional ‘hurdles’ ***Resource requirements*** • Required significant investment in effort/resources pre-operatively and post-operatively • Requirement for multiple stakeholder engagement; lack of multidisciplinary team involvement particularly in low resource hospitals • Poor quality food choices in hospitals ***Lack of ability to individualise content*** • Lack of ability to ‘tailor’ to individual patients if needed ***Other*** • Limited compliance, monitoring and feedback • Length of documented local ERAS protocols • Difficulties with representation / readmission if required (e.g., emergency length of stay, patients living further away, accommodation costs)	• Departmental/hospital-level implementation and standardisation, compliance monitoring and improvement feedback • Cross-departmental teams for implementation and education • Inclusion of a dedicated ERAS® manager or nurse for implementation and monitoring • Patient and family engagement • ‘Rebranding’ or naming of ERAS principles to part of standard care rather than a separate protocol • Include as a ‘foundation’ of informed consent

## Discussion

### Summary of results

This study has provided important baseline insights into the current state of ERAS perceptions across Australia and has described clinicians’ preferences for education and learning that can inform future ERAS implementation efforts. Overall, clinicians reported perceiving ERAS protocols in a largely beneficial light, with most also indicating they were ‘knowledgeable’ and had previously cared for a patient who was on an ERAS protocol. However, there were several key results and significant differences between professions that warrant further discussion.

### Adoption of ERAS protocols

All anaesthetist and most surgeon respondents indicated that they had previously participated in the care of a patient who was on an ERAS protocol. This is greater than the adoption found in a previous survey of colorectal surgeons and perioperative care in Australia and New Zealand, where 55% of 76 respondents did not care for patients using an ERAS protocol, 37% routinely did, and 8% did ‘sometimes’ [35]. More recently, researchers have reported ERAS implementation and/or use in Australian facilities [e.g. 36,37,38], along with the establishment of a local ERAS Centre of Excellence [22]. These resources, and our more contemporary results, suggest that ERAS uptake may be increasing.

It has been estimated that evidence takes up to 17 years to be translated into clinical practice [39], and support for the benefits of ERAS protocols has now been building for over a decade. The lag or gap between what is known and what is done, or the diffusion of innovation into the system and its members, has long been acknowledged as a challenge across many fields [40]. In other areas of health care, use of clinician practice guidelines may be lost across a four-stage pipeline: awareness, to agreement, to adoption, to adherence, and the simple ‘availability’ of a guideline does not result in complete uptake [26, 27]. To overcome the recognised evidence to practice gap [26, 39], translational efforts must support implementation into real-world contexts, particularly in the complex health care environment [41].

### Perceived knowledge about ERAS

Our results suggest that nurses’ perceived knowledge level was also lower than that reported by anaesthetists and surgeons. Nurses play a pivotal role across all phases of the patient’s surgical journey including contributing to and coordinating care directly related to ERAS components [42]. Their lack of awareness and knowledge of ERAS may suggest that protocols are often only partially or selectively implemented locally, which is a controversial approach to implementation not isolated to Australia [20, 21]. In a systematic review and meta-analysis of trials testing ERAS versus usual care for colorectal surgery in adults, protocols from 25 trials included a variation of between 4 and 18 elements [20]. Similarly, some of our survey respondents indicated that there is a need to be able to adapt interventions to individualise care. However, this contrasts with the multimodal, multidisciplinary principles of ERAS in which the intended approach is to include all elements as they have supporting evidence of improving patient outcomes [21]. The exclusion of certain evidence-based ERAS elements may limit patient benefit, and facilities should include as many as possible [21].

### Beliefs about ERAS

Respondents rated their beliefs around ERAS positively, although nurses less so, which may suggest limited knowledge rather than disagreement. Similarly, Beal and colleagues [32] found that most perioperative clinicians surveyed in their United States tertiary medical centre ‘strongly’ or ‘very strongly’ agreed that ERAS protocols were important for patient care, that their colleagues and administration felt the same, and that having patients involved in ERAS improved care. However, while our results indicate clinicians largely understand the benefits of ERAS overall, there are variations in support for ERAS.

In other areas, disagreement with clinical guideline components may result in failed adoption and adherence [26], and this may also limit ERAS uptake. A recent survey of Australian and New Zealand colorectal surgeons (*n* = 95) attitudes towards the effectiveness of 18 ERAS protocol components on short-term outcomes found that, for five interventions, 50–57% of surgeons felt they were ‘definitely’ or ‘very likely’ to be effective, but the remaining interventions had < 50% support to as low as 1–2% support [28]. Wide variations in implemented ERAS protocol components have been reported elsewhere [20], and there is a need to focus on the benefits of implementing ERAS protocols in their entirety in future research and educational strategies.

### Knowledge of ERAS: education and learning

Our survey also provided insights into the educational and learning preferences of respondents, which may be used to target and nuance implementation strategies. Our results suggest that Australian clinicians value patient-focused outcomes, with respondents most interested in learning about minimising perioperative complications versus least interested in improving perioperative efficiency (i.e., improving operational processes, while maintaining quality and safety, to optimise productivity and minimise costs and resource requirements [43]). Our results accord with Hughes and colleagues’ [44] multinational survey of patient and care provider attitudes to ERAS after major abdominal surgery across three centres in Scotland, Norway, and The Netherlands. All respondents rated outcomes highly on an 11-point Likert scale (0 not important to 11 very important; medians ≥ 7/11), with care providers and patients rating nausea control and the absence of pain at rest the most important. ERAS strategies were also rated highly, with preoperative counselling rating highly for providers and patients, and promoting and scheduling early mobilisation and avoiding hospital-acquired infection considered the most important for each cohort, respectively. However, higher support for all tested components contrasts with the results of Toh et al.’s [28] survey of Australian and New Zealand colorectal surgeons, where there was little support for the effectiveness of some. Given that some clinicians rated themselves as unknowledgeable about ERAS or commented that their colleagues had limited awareness of ERAS protocols in our survey, education is important and has been credited as a contributor to successful ERAS implementation elsewhere [45].

### Barriers and facilitators to implementation

ERAS protocols are complex to implement, given that full adherence involves the simultaneous implementation of *all* components and interventions among many healthcare providers across healthcare services [46]. While education is important, this alone will not bridge the knowledge to practice gap, and the effort required to appropriately implement ERAS protocols should not be underestimated [21]. Barriers identified in our survey are congruent with those experienced internationally [19], where resistance to change, limitation in resources, and external factors such as patient complexity and rural location have been highlighted.

In the Australian-based context, larger centre health services may face greater challenges in implementing ERAS compared to smaller single site facilities where there may be greater familiarity with surgical care pathways. Other barriers we have identified include the lack of interest and support from seniority and the inability to adapt content; similar to other older Australian studies [35, 47, 48]. Based on our own experience, gaining support from senior management to implement ERAS protocols into policy and source funding for coordinators and multidisciplinary support are significant barriers, while frontline clinicians are unable to be heavily involved due to workloads, despite their interest. Conversely, engaging support from clinicians and hospital leadership is a recognised facilitator for ERAS implementation, along with adaption to fit the local context, demonstrating early achievements, establishing a strong and regularly meeting ERAS team, and utilising ERAS supporters and dedicated staff [19]. These international facilitators are relevant to the Australian context and similar to those identified by our respondents, with the addition of compliance monitoring using quality indicators and improvement feedback, which is an important strategy for implementing and sustaining ERAS system-wide [49, 50].

From a patient perspective, our consumer investigators indicated they felt that implementation of ERAS was important for future patient care and safety. Inclusion of patients as stakeholders in future ERAS implementation and design is important to give patients agency in their own care, while patient experiences and perceptions shape the surgical care journey and influence compliance. Co-design with stakeholders, from senior management to patients, may be key to gaining insight into addressing these issues.

## Limitations

We acknowledge this study has several limitations. There may be some selection bias due to the sampling approach as only clinicians who were members of a professional college were invited to participate. Moreover, nurses were more highly represented than anaesthetists and surgeons in the sample, however all Australian states and two of three territories were represented. There may be limited external validity of our survey results beyond the specific sample. Further, it was not possible to calculate the response rate as precise membership numbers across the peak professional bodies fluctuate, therefore the accuracy of membership numbers at any time is variable. However, approximate membership numbers suggest the proportion who responded to our survey was small. As there was no individual or identifiable tracking of who received, opened, or completed the survey, there is a chance that clinicians could have provided multiple survey responses, but this is unlikely. Finally, there is the possibility of response bias due to social desirability, however, the anonymity measures that were used likely mitigated its effects, ensuring more accurate and reliable data collection.

## Conclusion

Our results suggest the need to promote ERAS use and provide education at a clinician and facility level. Our survey results also provide important insight into the preferred methods for learning about ERAS across professions. If clinicians have a better understanding of the principles and benefits of ERAS, they are more likely to advocate and use ERAS, leading to better patient outcomes. This is particularly relevant for nurses, who are well situated to contribute to and coordinate ERAS throughout the surgery journey [42], but who had lower knowledge levels and less experience with ERAS. While education will help in improving ERAS protocol knowledge and implementation, there is also a need to co-design implementation strategies with stakeholders that target identified facilitators and barriers.

### Electronic supplementary material

Below is the link to the electronic supplementary material.

Supplementary Material 1

## Data Availability

The datasets generated and/or analysed during the current study are not publicly available due to ethical and privacy restrictions but are available from the corresponding author on reasonable request.

## Abbreviations

ERAS: Enhanced Recovery After Surgery
