# Supplementary material for: Clinicians’ perceptions of “enhanced recovery after surgery” (ERAS) protocols to improve patient safety in surgery: a national survey from Australia
Source: Patient Saf Surg. 2024 May 23;18:18. doi: 10.1186/s13037-024-00397-w (PMC11119013; doi:10.1186/s13037-024-00397-w)
Supplement: Supplementary file 1 — Supplementary Material 1 [file 13037_2024_397_MOESM1_ESM.docx]

**Supplementary file 1: CROSS (Checklist for Reporting Of Survey Studies)**

|  | Item No | Recommendation | Page No |
| --- | --- | --- | --- |
| **Title and abstract** | 1 | (*a*) Indicate the study’s design with a commonly used term in the title or the abstract | 1 |
|  |  | (*b*) Provide in the abstract an informative and balanced summary of what was done and what was found | 2-3 |
| Introduction | | | |
| Background/rationale | 2 | Explain the scientific background and rationale for the investigation being reported | 3-5 |
| Objectives | 3 | State specific objectives, including any prespecified hypotheses | 5-6 |
| Methods | | | |
| Study design | 4 | Present key elements of study design early in the paper | 6-8 |
| Setting | 5 | Describe the setting, locations, and relevant dates, including periods of recruitment, exposure, follow-up, and data collection | 6-8 |
| Participants | 6 | (*a*) Give the eligibility criteria, and the sources and methods of selection of participants | 6, 8, Table 1 |
| Variables | 7 | Clearly define all outcomes, exposures, predictors, potential confounders, and effect modifiers. Give diagnostic criteria, if applicable | 6-9 |
| Data sources/ measurement | 8* | For each variable of interest, give sources of data and details of methods of assessment (measurement). Describe comparability of assessment methods if there is more than one group | 6-9 |
| Bias | 9 | Describe any efforts to address potential sources of bias | 17 |
| Study size | 10 | Explain how the study size was arrived at | NA |
| Quantitative variables | 11 | Explain how quantitative variables were handled in the analyses. If applicable, describe which groupings were chosen and why | 6-9 |
| Statistical methods | 12 | (*a*) Describe all statistical methods, including those used to control for confounding | 9 |
|  |  | (*b*) Describe any methods used to examine subgroups and interactions | 9 |
|  |  | (*c*) Explain how missing data were addressed | 9, 18 |
|  |  | (*d*) If applicable, describe analytical methods taking account of sampling strategy | NA |
|  |  | (*e*) Describe any sensitivity analyses | NA |
| Results | | | |
| Participants | 13* | (a) Report numbers of individuals at each stage of study—eg numbers potentially eligible, examined for eligibility, confirmed eligible, included in the study, completing follow-up, and analysed | 9 |
|  |  | (b) Give reasons for non-participation at each stage | 9, Figure 1 |
|  |  | (c) Consider use of a flow diagram | Figure 1 |
| Descriptive data | 14* | (a) Give characteristics of study participants (eg demographic, clinical, social) and information on exposures and potential confounders | 9-10, Table 2 |
|  |  | (b) Indicate number of participants with missing data for each variable of interest | All result tables, figure |
| Outcome data | 15* | Report numbers of outcome events or summary measures | 9-13 |
| Main results | 16 | (*a*) Give unadjusted estimates and, if applicable, confounder-adjusted estimates and their precision (eg, 95% confidence interval). Make clear which confounders were adjusted for and why they were included | NA |
|  |  | (*b*) Report category boundaries when continuous variables were categorized | 9-13 |
|  |  | (*c*) If relevant, consider translating estimates of relative risk into absolute risk for a meaningful time period | NA |
| Other analyses | 17 | Report other analyses done—eg analyses of subgroups and interactions, and sensitivity analyses | 9-13 |
| Discussion | | | |
| Key results | 18 | Summarise key results with reference to study objectives | 13-18 |
| Limitations | 19 | Discuss limitations of the study, taking into account sources of potential bias or imprecision. Discuss both direction and magnitude of any potential bias | 17 |
| Interpretation | 20 | Give a cautious overall interpretation of results considering objectives, limitations, multiplicity of analyses, results from similar studies, and other relevant evidence | 16-18 |
| Generalisability | 21 | Discuss the generalisability (external validity) of the study results | 17-18 |
| Other information | | | |
| Funding | 22 | Give the source of funding and the role of the funders for the present study and, if applicable, for the original study on which the present article is based | 19 |

*Give information separately for exposed and unexposed groups.

**Note:** An Explanation and Elaboration article discusses each checklist item and gives methodological background and published examples of transparent reporting. The STROBE checklist is best used in conjunction with this article (freely available on the Web sites of PLoS Medicine at http://www.plosmedicine.org/, Annals of Internal Medicine at http://www.annals.org/, and Epidemiology at http://www.epidem.com/). Information on the STROBE Initiative is available at www.strobe-statement.org.

**Supplementary file 2: GRIPP 2 (Guidance for Reporting Involvement of Patients and the Public) short-form checklist (Staniszewska et al., 2017)**

| **Topic** | **Item** | **Response** |
| --- | --- | --- |
| Aim | Report aim of PPI in study | - To clarify the aim of the survey, and to ensure it is important from the patient perspective - To include patient perspectives in the interpretations of the results - To identify any reporting items which require definition or elaboration |
| Methods | Provide a clear description of the methods used for PPI in the study | - Two consumers participated in the project as co-researchers - Both consumer researchers participated in an introductory phone meeting with the lead researcher - During the discussion of the protocol, consumer researchers did not suggest any changes, however, both consumers were not aware of the concept and use of ERAS protocols as part of the surgical care pathway. - One consumers contributed to the review of and comment on a summary of results to guide manuscript writing - One consumer provided written input into the manuscript |
| Study results | Outcome- report the results of PPI in the study (both positive and negative) | - Both consumers researchers believed that the topic area was important - Consumer comments on summary of results were used to:   - Guide the discussion and include discussion points   - Add definition of some items, elaboration of some results and discussion points - Amendments to the manuscript were included to represent the patient perspective |
| Discussion and conclusions | Outcome- comment on the extent to which PPI influenced the study overall (both positive and negative) | - PPI assisted in shaping the study and guide results interpretation and reporting, thus meeting the aims - Team members gained a better understanding of the importance of the patient perspective, and approaches to involving patients in research - Team members gained a better understanding of how to provide meaningful results to patients for comment and feedback |
| Reflections and critical perspective | Comment critically on the study, reflecting on the things that went well and those that did not so others can learn from this experience | - Prior to providing the results and manuscript to the consumers to review, we asked consumer researchers how they would like to receive the results and suggested a short summary for them to review and comment on. We felt asking our consumer researchers how they would like to provide input and review results would be beneficial and ensure they can use an approach that best suits them and their understanding. Both consumers accepted the suggested approach. - From the consumers perspective, I felt the communication was appropriate and tasks were negotiated well, ensuring I did not feel overburdened. |

PPI = Patient and Public Involvement

**Supplementary file 3: Survey instrument (after Beal et al., 2021; adapted and shared under CCBY-NC)**

| **NURSES** | **SURGEONS/ANAESTHETISTS** |
| --- | --- |
| ***Do you currently work in a setting that provided post-operative surgical care?***  Yes – survey continues ; No – survey end triggered | ***Not applicable*** |
| ***Please indicate the healthcare sector in which you generally work:***  Public, Private | |
| ***Location of your current facility:***  Australia – survey continues, Not practicing in Australia – survey end triggered | |
| ***City/Town of your facility:*** *Enter text* | |
| ***State/Territory/Region of your facility:*** *Enter text* | |
| ***What type of operating rooms are in your facility (Check all that apply):***  Outpatient surgery, Inpatient surgery, Adult surgery, Paediatric surgery | |
| ***Please select your department:***  Perioperative, Surgical ward/unit, 23 hour wards | ***Please select your department:***  Surgery, Anaesthesia |
| ***Not applicable*** | ***Please indicate your surgical/anaesthetic specialty:*** |
| ***Type of surgical teams/service you generally work in (Check all that apply):***  General Surgery, Gynecology, Urology, Orthopedics, Transplant, Trauma, Burns, Plastics, Ears Nose and Throat, Dental/Oral Surgery, Neurosurgery, Ophthalmology, Vascular, Cardiac, Thoracic, Other  ***If other, please specify:*** *Enter text* | |
| ***Please select your role:***  Circulating/Instrument RN, PACU RN, Surgical ward/unit RN, Nurse Unit Manager – Perioperative, Nurse Educator – Perioperative, Nurse Unit Manager - surgical ward, Nurse Educator - surgical ward, Other  ***If other, please specify:*** *Enter text* | ***Please select your role:***  Consultant Surgeon, Consultant Anaesthetist, Surgical Fellow, Anaesthetic Fellow, Registrar Surgeon, Registrar Anaesthetist, Non practicing academic surgeon, Non practicing academic Anaesthetist, Other  ***If other, please specify:*** *Enter text* |
| ***For how many years have you been practicing in your current role?*** *Enter text* | |
| ***Please select your gender:***  Male, Female, Non-binary, Prefer not to answer. | |
| ***Please indicate your age last birthday (in years):*** *Enter text* | |
| ***I have participated in the care of a patient who was on an ERAS protocol:***  Yes, No | |
| **PERCEPTIONS OF ERAS** | |
| ***Please indicate your knowledge level about ERAS protocols:***  Very Knowledgeable Knowledgeable Neutral Fairly unknowledgeable Very unknowledgeable | |
| ***Please select one: I believe ERAS protocols improve patient care.***  Strongly Disagree Disagree Neutral Agree Strongly Agree | |
| ***Please select one: I believe the hospital administration thinks ERAS protocols improve patient care.***  Strongly Disagree Disagree Neutral Agree Strongly Agree | |
| ***Please select one: I believe my colleagues think ERAS protocols improve patient care.***  Strongly Disagree Disagree Neutral Agree Strongly Agree | |
| ***Please select one: I believe that my patients have/will have improved care when they are involved in ERAS protocols.***  Strongly Disagree Disagree Neutral Agree Strongly Agree | |
| ***Please select one: I believe the enhanced recovery protocols are a reasonable investment of my time.***  Strongly Disagree Disagree Neutral Agree Strongly Agree | |
| ***Please select one: I believe that ERAS protocols improve/will improve the financial efficiency of our institution.***  Strongly Disagree Disagree Neutral Agree Strongly Agree | |
| **KNOWLEDGE OF ERAS PROTOCOLS** | |
| ***ERAS protocols are primarily designed to (please select one):***   - 1. Reduce the patient’s response to surgical stress to improve length of stay and reduce postoperative complications and mortality   2. Enhance the hospital’s efficiency and result in better financial outcomes for the hospital   3. Address patient expectations preoperatively to lead to improved patient satisfaction   4. All the above | |
| ***I am most interested in learning more about the following elements of ERAS (number in order 1 through 4)***  _Fluid management _Multimodal pain management _Minimizing perioperative complications _Improving perioperative efficiency | |
| ***My preferred method to learn about ERAS is:***   1. Direct participation in institutional protocols 2. Reviewing Journal articles or textbooks 3. Seminars or lectures on the topic from leaders at national or international levels 4. Seminars or lectures on the topic from leaders within my hospital or unit | |
| ***I think formal education about ERAS should be part of training for upcoming healthcare providers:***  ***Surgeons:*** Yes, No ***Anaesthestists:*** Yes, No ***Nurses:*** Yes, No | |
| ***I think barriers to gaining knowledge about ERAS include (choose all that apply):***  Lack of research, Lack of time, Lack of information provided, Lack of resources, Lack of interest from patients, Lack of interest from providers | |
| **FUTURE PLANNING** | |
| ***ERAS protocols should be implemented broadly:***  Yes, No  ***If yes, which patient populations:*** *Enter text* | |
| ***Please elaborate on any thoughts you have either for, or against ERAS protocols, as well as any general comments, considerations, or questions:***  *Enter text* | |

ERAS = Enhanced Recovery After Surgery; PACU = Post Anaesthetic Care Unit; RN = Registered Nurse

**Supplementary file 4: Beliefs about ERAS: Responses by agreement category**

|  | | **Overall**  ***n* = 178 (100%)** | **Anaesthetists**  ***n* = 26**  **(14.6%)** | **Surgeons**  ***n* = 36 (20.2%)** | **Nurses**  ***n* = 116 (65.2%)** |
| --- | --- | --- | --- | --- | --- |
| **I believe ERAS® protocols improve patient care* *n* (%)** | Strongly agree  Agree  Neutral  Disagree  Strongly disagree | 50 (32.3%)  69 (44.5%)  34 (21.9%)  2 (1.3%)  0 (0.0%) | 13 (52.0%)  12 (48.0%)  0 (0.0%)  0 (0.0%)  0 (0.0%) | 17 (53.1%)  12 (37.5%)  1 (3.1%)  2 (6.3%)  0 (0.0%) | 20 (20.4%)  45 (45.9%)  33 (33.7%)  0 (0.0%)  0 (0.0%) |
| **I believe the hospital administration thinks ERAS® protocols improve patient care***  ***n* (%)** | Strongly agree  Agree  Neutral  Disagree  Strongly disagree | 21 (13.7%)  55 (35.9%)  70 (45.8%)  7 (4.6%)  0 (0.0%) | 5 (20.0%)  10 (40.0%)  10 (40.0%)  0 (0.0%)  0 (0.0%) | 5 (16.1%)  11 (35.5%)  11 (35.5%)  4 (12.9%)  0 (0.0%) | 11 (11.3%)  34 (35.1%)  49 (50.5%)  3 (3.1%)  0 (0.0%) |
| **I believe my colleagues think ERAS® protocols improve patient* care *n* (%)** | Strongly agree  Agree  Neutral  Disagree  Strongly disagree | 14 (9.2%)  66 (43.1%)  66 (43.1%)  7 (4.6%)  0 (0.0%) | 4 (16.7%)  15 (62.5%)  3 (12.5%)  2 (8.3%)  0 (0.0%) | 5 (15.6%)  14 (43.8%)  10 (31.3%)  3 (9.4%)  0 (0.0%) | 5 (5.2%)  37 (38.1%)  53 (54.6%)  2 (2.1%)  0 (0.0%) |
| **I believe that my patients have/will have improved care when they are involved in ERAS® protocols* *n* (%)** | Strongly agree  Agree  Neutral  Disagree  Strongly disagree | 34 (21.9%)  80 (51.6%)  38 (24.5%)  3 (1.9%)  0 (0.0%) | 8 (32.0%)  15 (60.0%)  1 (4.0%)  1 (4.0%)  0 (0.0%) | 14 (43.8%)  10 (31.3%)  7 (21.9%)  1 (3.1%)  0 (0.0%) | 12 (12.2%)  55 (56.1%)  30 (30.6%)  1 (1.0%)  0 (0.0%) |
| **I believe ERAS® protocols are a reasonable investment of my time* *n* (%)** | Strongly agree  Agree  Neutral  Disagree  Strongly disagree | 42 (27.1%)  70 (45.2%)  41 (26.5%)  2 (1.3%)  0 (0.0%) | 9 (36.0%)  15 (60.0%)  1 (4.0%)  0 (0.0%)  0 (0.0%) | 15 (46.9%)  11 (34.4%)  5 (15.6%)  1 (3.1%)  0 (0.0%) | 18 (18.4%)  44 (44.9%)  35 (35.7%)  1 (1.0%)  0 (0.0%) |
| **I believe that ERAS® protocols improve/will improve the financial efficiency of our institution* *n* (%)** | Strongly agree  Agree  Neutral  Disagree  Strongly disagree | 39 (25.5%)  62 (40.5%)  47 (30.7%)  5 (3.3%)  0 (0.0%) | 6 (24.0%)  15 (60.0%)  4 (16.0%)  0 (0.0%)  0 (0.0%) | 16 (50.0%)  12 (37.5%)  3 (9.4%)  1 (3.1%)  0 (0.0%) | 17 (17.7%)  35 (36.5%)  40 (41.7%)  4 (4.2%)  0 (0.0%) |

*Note: ERAS = Enhanced Recovery After Surgery; IQR = interquartile range. Missing data* 12.9%, 14%, 14%, 12.9%, 12.9%, 14%*

**References**

Beal, E. W., Reyes, J. C., Denham, Z., Abdel-Rasoul, M., Rasoul, E., & Humeidan, M. L. (2021). Survey of provider perceptions of Enhanced Recovery After Surgery and perioperative surgical home protocols at a tertiary care hospital. *Medicine*, *100*(24), e26079. <https://doi.org.au/10.1097/MD.0000000000026079>

Sharma, A., Minh Duc N. T., Luu Lam Thang, T., Nam, N. H., Ng, S. J., Abbas, K. S., Huy, N. T., Marušić, A., Paul, C. L., Kwok, J., Karbwang, J., de Waure, C., Drummond, F.J., Kizawa, Y., Taal, E., Vermeulen, J., Lee, G. H. M., Gyedu, A., To, K. G., Verra, M. L., Jacqz-Aigrain, É. M., Leclercq W. K. G., Salminen, S. T., Sherbourne, C. D., Mintzes, B., Lozano, S., Tran, U. S., Matsui, M. & Karamouzian, M. (2021). A Consensus-Based Checklist for Reporting of Survey Studies (CROSS). Journal of General Internal Medicine, 36(10), 3179-3187. https://doi.org/10.1007/s11606-021-06737-1

Staniszewska, S., Brett, J., Simera, I., Seers, K., Mockford, C., Goodlad, S., Altman, D. G., Moher, D., Barber, R., Denegri, S., Entwistle, A., Littlejohns, P., Morris, C., Suleman, R., Thomas, V. & Tysall, C. (2017). GRIPP2 reporting checklists: Tools to improve reporting of patient and public involvement in research. *Research Involvement & Engagement, 3*(13). https://doi.org/10.1186/s40900-017-0062-2
